# Beliefs About the Speaker’s Reasoning Ability Influence Pragmatic Interpretation: Children and Adults as Speakers

**DOI:** 10.1162/opmi_a_00180

**Published:** 2025-01-20

**Authors:** Alexandra Mayn, Jia E. Loy, Vera Demberg

**Affiliations:** Department of Language Science and Technology, Saarland University, Saarbrücken, Germany; Department of Computer Science, Saarland University, Saarbrücken, Germany

**Keywords:** pragmatic inferences, partner effects, Rational Speech Act framework

## Abstract

The cooperative principle states that communicators expect each other to be cooperative and adhere to rational conversational principles. Do listeners keep track of the reasoning sophistication of the speaker and incorporate it into the inferences they derive? In two experiments, we asked participants to interpret ambiguous messages in the reference game paradigm, which they were told were sent either by another adult or by a 4-year-old child. We found an effect of speaker identity: if sent by an adult, an ambiguous message was much more likely to be interpreted as an implicature, while if sent by a child, it was a lot more likely to be interpreted literally. We also observed substantial individual variability, which points to different beliefs and strategies among our participants. We discuss how these speaker effects can be modeled in the Rational Speech Act framework.

## INTRODUCTION

If in response to the question “Do you have siblings?” someone were to say “I have a brother”, the likely interpretation would be that the speaker only has one male sibling, even though the statement is literally compatible with an interpretation where the speaker has two brothers, a brother and a sister, or more than two siblings. This is an example where the Maxim of Quantity, one of the maxims of cooperative communication famously formalized by the philosopher of language Paul Grice (1975), is operative. It states that, as a cooperative communicator, one should make their contributions to the conversation maximally informative, and saying that one has a brother when one in fact has two brothers would violate that principle.

An interesting question which can help to refine our theories of pragmatic processing is whether listeners have a generic model of the speaker, who they may or may not assume to be perfectly rational (i.e., to always select the optimal utterance given the meaning the speaker intends to convey) or whether they construct a speaker-specific model, whereby the characteristics of the specific speaker affect the interpretation. There is experimental evidence that in some situations the latter is the case: that people’s interpretations of the same utterances change depending on the identity of the speaker.

Beltrama and Schwarz (2021) showed that participants interpreted quantity expressions (prices and time) more precisely when they were uttered by a “nerdy” character than when they were uttered by a “chill” character. Schuster and Degen (2020) found that their participants picked up on differences in the use of uncertainty expressions such as “might” and “probably” by two speakers and derived a distinct interpretation depending on the speaker. Ip and Papafragou (2021) showed that the speaker’s language proficiency modulated the expectations that participants had of that speaker’s informativeness. While native speakers were thought to utter an underinformative sentence intentionally and were therefore deemed untrustworthy, when the same sentence was uttered by a not very proficient nonnative speaker of the language, their perceived trustworthiness was less affected, presumably because participants attributed the underinformativeness to limited language proficiency.

In the field of visual perspective taking, Duran et al. (2011) found that participants’ instructions for their conversation partner changed when they were made to believe that they were interacting with another participant as opposed to a simulated partner. Participants’ explanations became more egocentric, presumably because they ascribed greater cooperativity to human partners and expected them to shoulder more of the work in communication. In a more recent visual perspective taking study, Loy and Demberg (2023) also found that participants descriptions changed when they were interacting with a computer partner, but in the opposite direction than in Duran et al. (2011)’s study: participants behaved more egocentrically with a computer than with a human partner, and more egocentrically with a modern computer than with an older computer. The difference in the findings of these two studies may be explained by a shift in people’s perception of competence of artificial agents.

Grodner and Sedivy (2011) found that speaker reliability influenced the way scalar adjectives were interpreted. They showed that their participants interpreted scalar adjectives contrastively by default, as evidenced by a speedup in visually locating the referent when there was a contrasting item present in the display, but that this contrastive interpretation did not arise when participants were told that the speaker had an impairment “that caused language and social problems”.

The above studies showed that top-down information about the speaker, i.e., explicit mentions or visualisation of the speaker’s qualities, influences comprehension and inferencing. Gardner et al. (2021) and Ryskin et al. (2019) followed up on Grodner and Sedivy (2011) to investigate whether bottom-up information alone—observing the behavior of a speaker who uses scalar adjectives non-contrastively—could lead to the same effect on the interpretation as top-down information. They showed that, indeed, bottom-up information was sufficient to alter contrastive inferences given enough exposure.

In the current study, we add to this strain of work by investigating whether ambiguous messages will be more or less likely to be interpreted as an implicature as opposed to literally depending on the speaker’s perceived level of reasoning ability. For that, we use a pragmatic reference game paradigm based on the reference game introduced in Franke and Degen (2016). These types of reference games are frequently used for testing predictions of Rational Speech Act (RSA; Frank & Goodman, 2012) models and involve reasoning about messages and referents.

In two experiments, participants are asked to identify the referent of a message which, they are told, was sent to them by a participant of a previous experiment. We manipulate the identity of the speaker between participants: in one condition, it is a 4-year-old child from a local kindergarten, while in the other condition, it is an adult. We hypothesize that when the speaker is believed to be a child, ambiguous messages will be interpreted less pragmatically than when the speaker is believed to be another adult as we expect adults to have more uncertainty about whether 4-year-old children possess sufficient reasoning sophistication to act as pragmatic speakers.

Evidence that adults have beliefs about limitations of children’s communication and reasoning abilities comes from studies in which participants were explicitly asked to give an estimate of the age at which children acquire communicative abilities. Becker and Hall (1989) collected beliefs about the age of acquisition of communicative behaviors that rely on the understanding of context and appropriateness, such as appropriate turn-taking and explaining the reason for making a request. They found that for most of these skills, participants believed them to be acquired around the age of 6. Miller et al. (1980) presented their participants with a Piaget task battery, which assessed abilities including simple perspective taking based on asymmetric perspective, as well as understanding of physical properties of objects such as conservation of weight and height, and asked them to estimate at what age children acquire these abilities. The average estimated ages of acquisition of the two perspective-taking tasks, which involved realizing that the person sitting opposite of the child sees a picture from a different perspective, were 4.30 and 5.11 years. These findings suggest that adults have beliefs about pragmatic and communicative competence of a child at a particular age and that there would be uncertainty about a child’s pragmatic competence at the age of 4. However, these studies asked participants about a child’s abilities explicitly, whereas in our study, we ask whether adults will deploy their beliefs about children when interpreting signals purportedly sent by them.

Indeed, we find an effect of speaker identity: if the speaker is believed to be another adult, an ambiguous message is more likely to be interpreted as an implicature, that is, as intentionally uttered and carrying more than just the literal meaning. On the other hand, if the speaker is believed to be a child, the message is more likely to be interpreted literally. However, we also observe substantial individual variability, suggesting that participants have varying beliefs about reasoning complexity of children and adults and that participants themselves vary in their reasoning complexity.

There appears to be both a categorical and a gradient component to observed effect: more people appear to be deriving an inference in the adult speaker condition, and participants in the adult speaker condition tend to derive stronger inferences than participants in the child speaker condition.

We discuss the implications of these findings for the theories of perspective taking and for computational modeling of perspective taking in the RSA framework.

## BACKGROUND: RATIONAL SPEECH ACT MODELS

Grice’s principles of cooperative, or rational, communication have been formalized in probabilistic models of pragmatics, such as the Rational Speech Act model (Frank & Goodman, 2012). The Rational Speech Act model (RSA) is a Bayesian model where a speaker and a listener recursively reason about each other and possible alternative utterances and meanings. It has been successfully applied to modeling a variety of nonliteral language use phenomena, such as scalar implicatures (Goodman & Stuhlmüller, 2013), hyperbole (Kao, Wu, et al., 2014) and metaphor (Kao, Bergen, et al., 2014).

A paradigm which is often used for testing the predictions of RSA models is the reference game (e.g., Frank, 2016; Frank & Goodman, 2012). We use a version of this paradigm in the current study. A reference game involves two players, a listener and a speaker. The speaker’s task is to send the listener a message to refer to one of the objects in the shared view. The listener’s task, in turn, is to identify the referent of the speaker’s message. Often the set of messages the speaker can use is restricted. Since RSA is a model of cooperative interaction, it is assumed that the speaker will always say things that are literally true.

**Figure 1.**
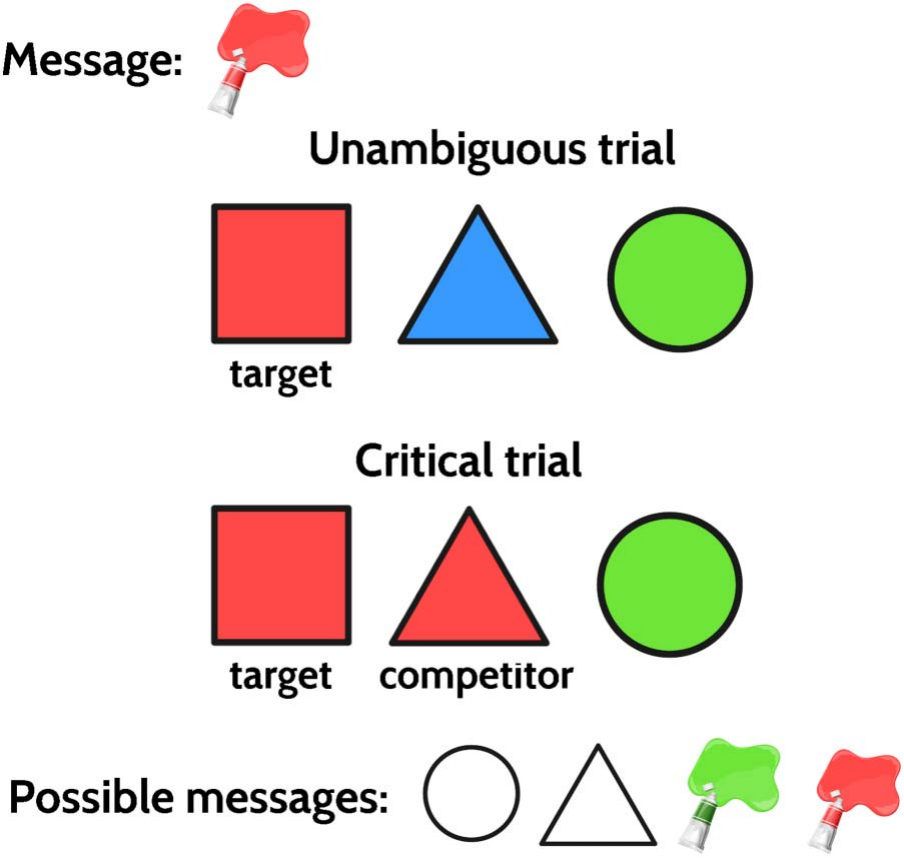
Examples of an unambiguous and an implicature reference game trials.

An example of a reference game listener trial from our experiment, which we will use to explain various RSA model flavors, is presented in Figure 1. In the unambiguous trial, the message “red” uniquely identifies the referent, red square, since that is the only red object. In the critical, or implicature, trial, however, the message is at first glance ambiguous since there are two red objects. In order to decide which of the two red objects the speaker meant to refer to, the listener needs to perform pragmatic reasoning about alternatives that were available to the speaker. The red triangle could also be referred to using the triangle message, whereas the message “square” is not available, and thus the only way to refer to the red square is the message “red”. Therefore, the listener should pick the red square over the red triangle upon hearing “red”. We will call the red square the target and the red triangle the competitor. Picking the target means correctly solving the implicature, i.e., resolving the ambiguity by reasoning about the speaker’s intended meaning. A literal interpretation of a message on an implicature trial is therefore ambiguous between the target and the competitor, whereas a pragmatic interpretation favors the target as a result of reasoning about alternatives the speaker could have used.

In the Rational Speech Act model, listener *L*_*N*_ and speaker *S*_*N*−1_ are Bayesian agents who recursively reason about each other and alternative possible utterances by the speaker and interpretations by the listener. Models of different recursion depth make different predictions for the performance on the reference game.

In RSA, the most unsophisticated listener model is *L*_0_, a so-called literal listener. *L*_0_ does not take the speaker’s perspective into account and simply assigns equal probability to all referent objects *o* of which the utterance *u* is literally true. The probability distribution of the literal listener is defined as *L*_0_(*o*∣*u*) ∝ [[*u*]] · *P*(*o*), where [[*u*]] is the boolean function of the utterance’s meaning, corresponding to whether it is literally true of the object *o*, and *P*(*o*) is the prior probability that the object will be referred to. *P*(*o*) is often defined as the object’s salience. Since *L*_0_ interprets utterances literally, it will correctly identify the target on the unambiguous trials but not on the critical ones. In the literature, *L*_0_ is sometimes viewed not as an actual model of a conversational agent but as a dummy component of more complex RSA models that grounds the meaning of utterances and gets recursion off the ground (Degen et al., 2020). However, Franke and Degen (2016) found that some participants make choices consistent with predictions of an *L*_0_ model and can therefore be considered literal listeners.

*L*_2_ is a pragmatic listener model that is most commonly used in RSA modeling literature.^1^ *L*_2_ reasons about the pragmatic speaker *S*_1_, who, in turn, reasons about the literal listener *L*_0_. *S*_1_ is defined as *S*_1_(*u*∣*o*) ∝ *exp*(*α* · (log *L*_0_(*o*∣*u*) − *Cost*(*u*)). The speaker seeks to maximize their informativity, that is, the probability that the literal listener *L*_0_ will correctly identify the intended referent, while minimizing utterance cost. For the example in Figure 1, *S*_1_ will prefer the message “triangle” to refer to the red triangle, and the message “red” to refer to the red square. The speaker model also includes the temperature parameter *α*, which controls the degree to which the speaker maximizes their utility, with target probability approaching 1 as *α* grows. As *α* approaches 0, the speaker approaches a literal speaker who is equally likely to produce any literally true message. Finally, *L*_2_ is then defined as *L*_2_(*o*∣*u*) ∝ *S*_1_(*u*∣*o*) · *P*(*o*), where *P*(*o*), like in *L*_0_, is the prior probability of the object being referred to. It mirrors the definition of *L*_0_, with the speaker probability distribution instead of the literal meaning.

Those are the models traditionally used in the RSA literature, but other models can be defined. Franke and Degen (2016) defined a model they termed *exhaustive listener L*_1_ as a separate model that reasons about the literal speaker *S*_0_, who is equally likely to produce every utterance that is true of the referent. For the example in Figure 1, *S*_0_ will assign equal probability to the red square and the red triangle since the message “red” is literally true of both of them.

In theory, more levels of recursion are also possible. However, those models are unlikely to be plausible models of human reasoning (Frank, 2016).

Now, how do these models map onto the predictions we make for our experiments?

We hypothesize that, at the population level, participants will be more likely to interpret the message pragmatically when the speaker is an adult than when it is a child. When the speaker is a child, we expect participants to be more likely to not derive an implicature or to derive a less strong implicature because they may believe the child’s reasoning ability to be insufficient for selecting the optimal message.

The effect of speaker identity on inferences can be modeled as listener’s beliefs about the speaker’s recursion depth. When faced with a speaker, the listener will be uncertain about whether they are dealing with a literal speaker *S*_0_ who sends a literal message, or a pragmatic speaker *S*_1_ who always chooses optimally. The listener’s beliefs about the reasoning depth of the speaker they are dealing with is expressed with the weight *λ*:

$$L_{2}^{\text{belief}-\text{driven}}\left(o|u\right)\propto S_{\text{weighted}}\left(u|o\right)\cdot P\left(o\right)$$

$$\text{where}\;S_{\text{weighted}}\left(u|o\right)\propto \lambda \cdot S_{1}\left(u|o;\alpha \to \infty \right)+\left(1-\lambda \right)\cdot S_{0}\left(u|o\right)\;\text{and}\;0\leq \lambda \leq 1$$

If *λ* = 1, the listener is confident that the speaker is fully rational and always selects the optimal utterance. At *λ* = 0, the listener is confident that the speaker is literal and randomly selects a true message to send to refer to an object. For in-between *λ*-values, the listener has some uncertainty about the speaker’s rationality. Derivation of the model’s predictions for different *λ* values is included in the Appendix.

The mixture mechanism in the proposed implementation is inspired by the rational mixture mechanism of rational spatial perspective-taking (Hawkins et al., 2021) but here it has a different interpretation. In Hawkins et al. (2021)’s model, the mixing weight *w* is shared between two speaker perspectives, egocentric and allocentric, and the speaker chooses how heavily to weigh each of the perspectives based on an informativity-cost tradeoff. Here, the mixing weight *λ* reflects the *listener’s* existing beliefs and potential uncertainty about the recursion depth of the speaker. Our proposed use of a mixture weight to represent uncertainty is closest to Schuster and Degen (2020)’s model of uncertainty expressions, whose expected pragmatic speaker model is a weighted average over different speaker models which differ in terms of thresholds and cost functions.

We expect that individual participants will vary in terms of their beliefs, which can be expressed with different settings of *λ*, but also that participants will on average interpret messages uttered by an adult more pragmatically, corresponding to a higher *λ* in the adult speaker condition.

When *λ* = 1, reflecting full listener certainty that they are dealing with a rational speaker, $L_{2}^{\text{belief}-\text{driven}}$ (*target*∣*u*) will be 1 as well, whereas when *λ* = 0, $L_{2}^{\text{belief}-\text{driven}}$ will essentially be equivalent to an *L*_1_ model and $L_{2}^{\text{belief}-\text{driven}}$ (*target*∣*u*) = $\frac{2}{3}$, and for intermediate *λ* values, the probability assigned to the target will be between $\frac{2}{3}$ and 1. Let us explain why $L_{2}^{\text{belief}-\text{driven}}$(*target*∣*u*; *λ* = 0) is $\frac{2}{3}$ and not $\frac{1}{2}$. It is because the RSA assumes that the pragmatic listener is rational and, in addition to reasoning about the speaker rationality, also reasons about alternative utterances. Let’s again consider the example in Figure 1. If *S*_0_ wants to refer to the red square, they will use the message “red” 100% of the time since that is the only literally true message. If they want to refer to the red triangle, they will choose the message “red" half of the time and the message “triangle” half of the time. Therefore, *L*_1_ reasons that if the message “red" is used, it is twice as likely to be referring to the red square. Hence the model predicts that, even in the child speaker condition, when the listener is convinced that the child is fully literal, the $L_{2}^{\text{belief}-\text{based}}$ model will assign the probability of at least $\frac{2}{3}$ to the target. We will return to how well this property of the model is supported by our data in discussion.

While we expect our participants at the population level to reason about the speaker and the speaker’s reasoning complexity, as Franke and Degen (2016) found, some participants seem to interpret messages literally and do not take the speaker’s perspective into account at all, as described by the literal *L*_0_ model. *L*_0_ predicts that those participants will assign equal probability to the target and the competitor. We expect that there will be some such participants but, since participants are assigned to conditions randomly, there should be a roughly equal number of them in both conditions, which means that we should still be able to detect differences between conditions.

## EXPERIMENT 1

This experiment investigated the effect of speaker identity on the interpretation of ambiguous messages.

In a between-subjects design, participants were told they would interpret pictorial messages sent either by another adult or by a four-year-old child and indicated their beliefs about the intended referent by distributing 100 points between 3 objects—target, competitor and distractor—using sliders. On critical trials, the message is ambiguous between the target and the competitor, if taken literally. However, the ambiguity can be resolved by reasoning that there is only one way of referring to the target because a message for the other feature of the target was not available to the speaker, whereas both features of the competitor (shape and color) were expressible as messages.

We hypothesized that there would be an effect of speaker identity: in the adult speaker condition, we expect participants to interpret ambiguous messages more pragmatically than in the child speaker condition, resulting in higher ratings assigned to the target on critical trials. Additionally, it is possible that target ratings in the unambiguous condition, where the message only matches the target, are higher in the adult condition as well if participants have some uncertainty about the child being able to reliably select a message that matches one of the features of the referent even in the unambiguous context.

### Participants

80 native speakers of English, 40 per speaker condition, with an approval rating of at least 95% were recruited via the crowdsourcing platform Prolific. 6 participants (3 in the adult condition, 3 in the child condition) were excluded because their average target rating on the unambiguous trials was below 80, suggesting that they may have misunderstood the task or randomly clicked through the experiment. 3 participants (1 in the adult condition, 2 in the child condition) were excluded because their reported strategy suggested that they had misunderstood the setup of the experiment. 1 participant in the adult condition was excluded because they had had a technical issue. New participants were recruited in their place, resulting in 40 participants in the adult speaker condition and 39 in the child speaker condition. There are 39 and not 40 participants in the child speaker condition because we excluded an additional participant upon closer inspection of the data once we’d already finished data collection. Excluding this participant from the analysis or including them does not affect any of the results.

### Design

#### Materials.

The paradigm we used in the experiment is a reference game based on Experiment 4 of Mayn and Demberg (2023), which, in turn, is based on Experiment 1 of Franke and Degen (2016), with some modifications. Participants’ task was to guess the referent of a message which they were told had been sent to them by a participant of a previous study (the speaker). There were three possible referents, each of which was composed of two features, a shape and a color. Each of these referents had one of three shapes (square, circle or triangle) and one of three colors (blue, red or green). The message that the participants received on each trial was a shape or a color. However, participants were told that not all colors and shapes were expressible as messages: there were no messages for the square or for the color blue. Those are therefore considered *inexpressible* features.

Participants’ task was to indicate for each of the three objects how likely they thought it was the object that the speaker was trying to get them to pick out. Participants responded by distributing 100 points between the three objects using sliders.

The experiment consisted of 24 trials, of which 8 were critical and the remaining 16 were fillers. Each trial display consisted of the target, competitor and distractor, presented in random order. Trial order was randomized for every participant.

**Figure 2.**
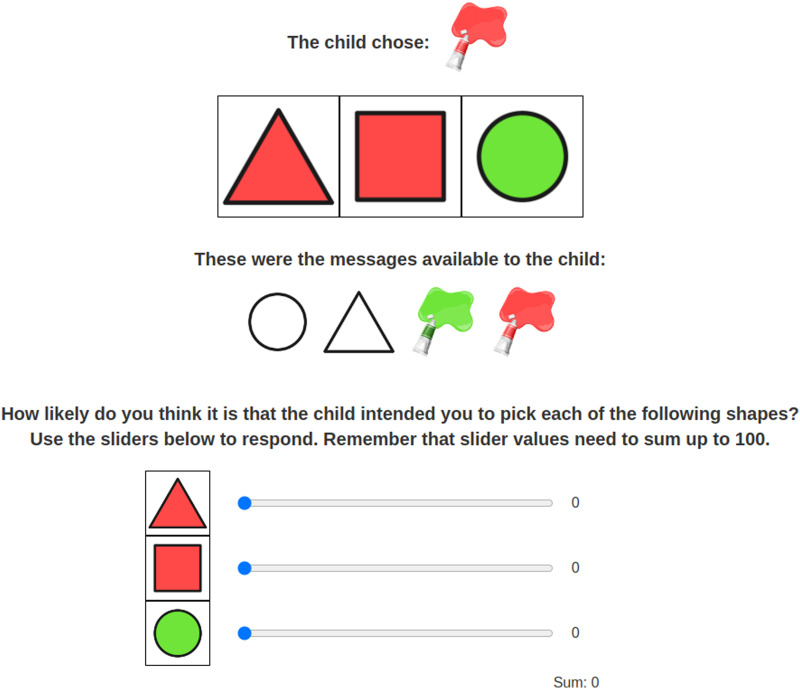
An example of a critical trial in the child speaker condition.

On critical trials, the message was ambiguous. An example of a critical trial is presented in Figure 2. The message (the color red), if taken literally, matches two of the objects—the square and the triangle. However, one may resolve the ambiguity by reasoning about the possible alternative utterances the speaker could have used. If the speaker had meant to refer to the red triangle, they had an unambiguous message available to them (the triangle). Since the speaker chose not to use this message, one may reason that the speaker meant to refer to the red square since there is no unambiguous way to refer to that object as “square” is not an available message. Therefore, the red square is the target and the red triangle is the competitor in this case. However, one may also either fail to or decide not to draw that implicature and interpret the message literally. This study aims to explore whether speaker identity may influence whether the listener may not derive the implicature.

Of the 16 fillers, 4 are completely unambiguous, where the features of every object are unique, and 4 are completely ambiguous, meaning that they contain two identical objects which are equally likely to be the referent. The remaining 8 trials are unambiguous and have the same display as the 8 critical trials but the target is the competitor from the corresponding critical item (4 trials) or the distractor from the corresponding critical item (4 trials).

The critical trials in this experiment correspond to the simple condition in Franke and Degen (2016). Their study additionally included “complex” trials, where, unlike in simple condition, the competitor had a feature in common with the distractor. They showed that participants indeed performed worse on the complex trials compared to the simple trials. We decided to only use the simple condition in this study for the following reason. Our hypothesis is that listeners may perform meta-reasoning about the reasoning complexity of the speaker, which may then influence the listener’s interpretation. However, if the task is too complex for the listener to solve, they are unlikely to additionally engage in meta-reasoning. Therefore, we only use simple implicature trials for this task which participants have been shown to be more successful at solving. While the task in Franke and Degen (2016) was a forced choice task between the three possible referents, in our experiment, we ask participants to distribute 100 points between the three referents with the aim of collecting more informative graded judgements.

#### Speaker Identity Manipulation.

The critical manipulation was the identity of the speaker whose messages participants were asked to interpret. Participants were randomly assigned to either the adult speaker or the child speaker condition. In the adult speaker condition, we told participants that the messages were sent by a participant of a previous experiment. In the child speaker condition, we used the cover story that we had recently conducted a study with 4-year-old children at a local kindergarten. In Experiment 1, the cover story included children being told that they were scientists communicating with an alien and selecting a message to send to the alien so that she alien could identify the correct object. We told participants in that condition that they would be interpreting messages that one of the children chose to send to the alien. In Experiment 2, we made the child speaker condition more comparable to the adult speaker condition by removing the mention of the alien and instead including a photo of a 4-year-old child.

We hypothesized that listeners may not derive an implicature or derive a less strong implicature if they believe that the speaker’s reasoning ability may be insufficient for determining what the optimal message to send is. We hypothesized that participants would assign lower probability to the target in the child speaker condition because they would think that a 4-year-old’s reasoning may not be sophisticated enough to reason that, if there are two messages that apply to the target and one of them is ambiguous while the other one is unambiguous, the unambiguous message is preferable.

### Procedure

At the start of the experiment, participants briefly, for 3 trials (two completely unambiguous and one completely ambiguous), took the perspective of the speaker. This was done in order to make sure that participants understood the relationship between the messages and the referents and the fact that not all messages were available to the speaker.

Participants then completed the 24 experimental trials. Finally, each participant saw the first critical trial they had seen in the experiment again and, once they had given a response using sliders, the sliders were disabled and the question “Why did you decide to put the sliders in those positions?” appeared on the screen next to the sliders along with a text box. We included this open-answer question, following Mayn and Demberg (2023), to get an additional insight into how participants went about solving the task. Additionally, we are interested in whether the meta-reasoning we are expecting is a conscious process. If so, we would expect it to be reflected in participants’ explanations.

The whole experiment took participants about 10 minutes to complete.

### Annotation of Reasoning Strategies

We annotated participants’ responses to the question “Why did you decide to put the sliders in those positions?” using the annotation scheme from Mayn and Demberg (2023). The category *correct_reasoning* was assigned to responses describing hypothetical reasoning about alternatives. Random guessing between the competitor and the distractor was assigned to the category *guess*. Responses which indicated that a participant chose an object due to a preference for one shape or color over another, or because the shape or the color stood out more, were assigned the tag *salience*/*preference*. Preference is closely related to guessing but, unlike guessing, it is more likely to result in consistent choices. Answers where it was not clear what the participant meant or answers which did not reveal anything about the participant’s strategy were labeled *unclear*. Answers which indicated that the participant changed their mind were labeled *changed_mind* and were not considered in the analyses of annotations since they indicated that the participant’s post hoc reasoning did not match their reasoning in the moment.

In a few cases, a participant’s reported strategy indicated that they had misunderstood the instructions of the experiment. They took the fact that a feature is inexpressible (e.g., that there’s no message “square”) to mean that an object with that feature cannot be referred to. If a participant’s answer indicated that they had misunderstood the instructions, the reported strategy was assigned the tag *misunderstood_instructions* and the participant was excluded from all analyses, and a new participant was recruited in their place.

There are other tags in Mayn and Demberg (2023)’s annotation scheme but we do not describe them here as no instances of them occurred in our data.

In addition, we introduced one tag which was not present in Mayn and Demberg (2023)’s annotation scheme: *meta_reasoning*. It was assigned when the answer included reasoning about the alternative messages available to the speaker, as well as explicitly stated uncertainty about whether the speaker would have been capable of selecting the optimal message. An example from the child speaker condition is “I am in two minds if a 4-year-old would be smart enough to choose the triangle shape if the target is the red triangle, rather than the color red.”

Example responses for each annotation tag are reported in Table 1. The annotation scheme with more examples for each annotation tag can be found in the Appendix, and the whole annotated dataset is included in the repository with data and scripts.

**Table 1.** Annotation scheme which was used to label participants’ explanations.

Annotation tag	Example
correct_reasoning	If the speaker had wanted to refer to the triangle, they could have used the message triangle, which would have been unambiguous.
meta_reasoning	I am in two minds if a 4-year-old would be smart enough to choose the triangle shape if the target is the red triangle, rather than the color red.
guess	There are 2 red objects, so it’s 50-50.
salience/preference	The triangle may be more popular.
unclear	This is what I would have meant if I were the speaker.
changed_mind	I picked the triangle but now I think it’s the square.
misunderstood_instructions	The square was not available so the triangle must be the target.

It should be noted that even if a participant’s open-ended answer does not include meta-reasoning about the speaker, that does not necessarily mean that no meta-reasoning occurred. It may be that either this meta-reasoning is not entirely conscious, at least for some people, or they engaged in meta-reasoning but did not report it.

### Results

**Figure 3.**
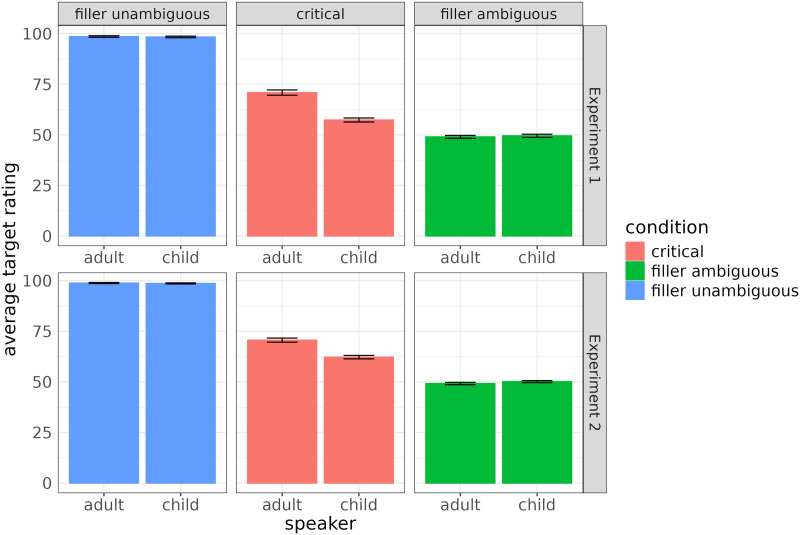
Average target ratings in the two speaker conditions for all trial types.

First, we look at the average probability (out of 100 points) assigned to the target for the unambiguous, ambiguous and critical trials. Top panel of Figure 3 shows that in both the child and the adult speaker conditions, participants’ performance was at ceiling on unambiguous trials and at chance between the two identical options on ambiguous ones. This strongly suggests that participants understood the task and that participants judged 4-year-olds to be capable at least of feature matching. Accuracy on ambiguous trials is at chance because on those trials, there are two identical objects and a coin is flipped to decide which of them is the target. On critical trials, average target probability is 70.8 (*SE* = 1.3) in the adult speaker condition and 57.3 (*SE* = 0.98) in the child speaker condition. This suggests that participants indeed tend to be more likely to draw the inference if they believe that the speaker’s reasoning is sufficiently sophisticated to select the optimal message.

To verify this apparent effect, we fit a Bayesian regression model to the data using the *brms* package in R. For this analysis, we only used critical and unambiguous trials because ambiguous trials were mainly included for counterbalancing purposes and do not pertain to the question of interest. We regressed the probability assigned to the target onto the speaker condition (sum-coded, levels: −1 = child, 1 = adult), trial type (sum-coded, levels: −1 = unambiguous (control), 1 = critical), an interaction between speaker condition and trial type, target position (three-level factor: left, middle, right; dummy-coded with middle as reference), trial number (mean-centered), and message type (shape or color; sum-coded, levels: −1 = shape, 1 = color).

The random effect structure included per-participant and per-item random intercepts as well as per-participant random slopes for trial type, message type and trial number.

For all effects, wide weakly informative priors were set. Specifically, for the effects of interest—the effect of speaker condition and interaction of speaker condition with trial type—we intentionally set very wide priors to avoid biasing the model in any direction. For the speaker condition, we set the prior to be a normal distribution centered around 0 with a standard deviation of 12.5 so that the whole probability range 50–100 (50 corresponding to literal responding and guessing between target and competitor and 100 corresponding to perfectly rational responding) is covered within 2 standard deviations. The same wide prior of a normal distribution centered around 0 with a standard deviation of 12.5 was applied to the interaction of speaker condition and trial type. The full model equation as well as a detailed discussion of all priors is included in the Appendix. We ran four chains of 4000 iterations each, with the first 1000 iterations of each chain discarded as warm-up.

To assess the evidence for the effects, we examined whether the credible intervals for the parameter estimates included zero. If the credible interval did not include zero, we concluded that there was a meaningful relationship between the predictor and the dependent variable. The results are reported in Table 2. Unsurprisingly, much lower target ratings were assigned on critical trials compared to unambiguous trials ($\hat{\beta }$ = −17.25, 95% CrI = [−19.18, −15.27]). There is a very small but robust effect of trial number, suggesting that participants on average became slightly better over the course of the experiment ($\hat{\beta }$ = 0.14, 95% CrI = [0.03, 0.24]).

**Table 2.** Effect estimates and 95% credible intervals (CrI) for the two experiments. Effects for which the 95% CrI does not include 0 are boldface.

**Effect**	**Experiment 1 (*N* = 79)**		**Experiment 2 (*N* = 160)**	
	**Estimate**	**95% CrI**	**Estimate**	**95% CrI**
**Intercept**	81.81	[79.63, 84.03]	82.67	[81.07, 84.22]
**Speaker (adult vs. child)**	3.37	[1.41, 5.34]	1.90	[0.41, 3.39]
**Trial type (critical vs. control)**	−17.25	[−19.18, −15.27]	−16.19	[−17.62, −14.79]
**Trial number**	0.14	[0.03, 0.24]	0.17	[0.10, 0.24]
Message type (color vs. shape)	0.51	[−0.16, 1.19]	−0.03	[−0.46, 0.40]
Targetpos (left vs. center)	−0.48	[−1.61, 0.69]	0.34	[−0.60, 1.27]
Targetpos (right vs. center)	−1.14	[−2.27, 0.00]	−0.67	[−1.60, 0.25]
**Speaker : Trial type**	3.17	[1.35, 5.00]	1.83	[0.42, 3.27]

Crucially, participants assigned higher probability to the target in the adult speaker condition than in the child speaker condition ($\hat{\beta }$ = 3.37, 95% CrI = [1.41, 5.34]). There was also an interaction of speaker condition with trial type, whereby the difference in target ratings was larger on critical trials than on unambiguous ones ($\hat{\beta }$ = 3.17, 95% CrI = [1.35, 5.00]). Therefore, we can conclude that speaker’s identity and perceived reasoning complexity influences the interpretation of ambiguous messages.

Next, we take a look at the annotations of participants’ strategies. In the adult speaker condition, there’s a much higher proportion of *correct_reasoning* responses (17 out of 40, 43.6%) than in the child speaker condition (6 out of 39, 15.4%). Interestingly, even in the adult condition, there were quite a few guesses (16 out 40, 41.1%, vs. 23 out of 39, 59%, in the child speaker condition). This suggests that at the population level, people are more likely to derive an implicature in the adult condition but some individuals do not do so. This is not surprising given Franke and Degen (2016)’s original findings that participants could be divided into reasoning complexity types based on their performance and some participants fell into the literal listener (*L*_0_) reasoning type. If someone is not able to reason pragmatically themselves, they will also not be able to meta-reason about whether the speaker is able to do so.

In both conditions, there are a few *meta_reasoning* responses (4, 10.3%, in the adult and 5, 12.8%, in the child speaker condition), suggesting that people are sometimes aware of deriving a less strong implicature based on considerations about the speaker. We take a closer look at reasons that were named for providing a lower target rating in the explanations in the *meta_reasoning* category. In the adult speaker condition, the reasons were the following: “some allowance for the participant thinking illogically or mistakenly”, “I think some people may miss this”, “there is a small chance they didn’t think of that” and “it can’t be fully ruled out [that the speaker meant the competitor]”. In the child speaker condition, the following reasons were mentioned: “I think there’s a chance (25%) that the child operated entirely off color”, “There’s some doubt [that the child selected the message pragmatically]. So I accounted for that not very likely possibility”, “There’s a chance they were just picking by colour without making that calculation”, “I think there is a 70% chance that the child would be smart enough to do that”, and “A child’s thought may not follow this process, hence the 20% error”. It is noteworthy that in the adult condition, too, some participants acknowledge the possibility of error or not paying attention resulting in non-optimal responding. Another interesting observation is that in some of these explanations, people directly refer to the provided ratings and describe the rating assigned to the competitor as the chance that the speaker was not thinking optimally (e.g., 25% chance). From the perspective of probability theory, this is not correct since even the literal speaker will select the pragmatically optimal message half of the time by chance. This kind of probabilistic reasoning errors has been widely discussed for other phenomena (e.g., Cohen & Staub, 2015; Fox & Levav, 2004; Saenen et al., 2018; Stengård et al., 2022), as well as recently for the reference game paradigm (Mayn et al., 2024). We will return to potential probabilistic reasoning errors in the current study in the discussion.

There is only one response in the *salience*/*preference* category, in the child speaker condition, which is “the red option may be more popular [than the blue option]”. The fact that there was only one explanation falling into this category, together with the fact that there was no effect of message type (color vs. shape) in the regression model, suggests that salience did not have a large effect on participants’ responses. However, even if salience did have a small effect, we assume that it would affect participants in the two speaker conditions in the same way.

It is highly unlikely that there were a lot more literal reasoners in the child speaker condition due to chance. Instead, it seems to be more likely that some of the *guess* responses in the child speaker condition involved not deriving the implicature because of considerations about the child’s reasoning ability but either the participants were not aware of the meta-reasoning they were performing or they did not report it in their explanations.

Additionally, when examining the average target probability associated with each annotation tag, we see that, both in the case of *correct_reasoning* and *meta_reasoning*, the associated target probability is higher in the adult speaker condition than in the child speaker condition (88.0 (*SE* = 3.43) vs. 73.8 (*SE* = 6.82) for *correct_reasoning* and 87.5 (*SE* = 5.7) vs. 72.0 (*SE* = 3.14) for *meta_reasoning*), suggesting that even when people believe that the child might be able to solve the task, they are less certain, and in the cases where participants explicitly reason about the speaker’s reasoning sophistication, they assign only a small probability to an adult not thinking optimally and a larger probability to a child not thinking optimally.

Since this experiment’s sample size is not very large, we cannot draw definitive conclusions based on the differences in annotation numbers. Instead, we view this analysis as supplementary to the main regression analysis, and since the annotations of reasoning strategies point to the same conclusions as the regression analysis, we view them as additional support for our findings.

Taken together, the results suggest that, provided that the listener themself has sufficient reasoning ability, they will take the reasoning ability of the speaker into account and may not derive an implicature or derive a weaker one if they believe the speaker’s reasoning ability to be insufficient for selecting the optimal message.

### Discussion

This experiment provided evidence that, at the population level, people take incorporate their beliefs about the speaker’s reasoning ability into the inferences they derive. On critical trials, participants were more likely to derive an inference if they believed the speaker was an adult, as indicated by higher target ratings.

The effect that we reported is a population-level effect. Top panel of Figure 5 displays individual participants’ average target ratings and shows that there is a lot of individual variability. The effect appears to be driven by two factors: participants in the adult condition assigning higher ratings to the target, corresponding to drawing a stronger inference, and more participants in the child speaker condition assigning a 50% rating to the target, corresponding to not drawing an inference.

An anonymous reviewer pointed out a potential confound in the setup of the experiment: in the child speaker condition, the cover story involved the children being told to imagine that they are scientists communicating with an alien. Our intention in constructing the instructions that way was to make the cover story believable by presenting the instructions in the format of a game that would be accessible and engaging for children. However, as the reviewer rightly pointed out, this may have had unintended effects related to the perception of aliens. Participants’ lower target ratings in the child speaker condition may have reflected not their beliefs about children but their beliefs about aliens: aliens may not be perceived as pragmatic communicators, and one may communicate differently with an alien than with another human.

In order to address this concern, we conducted a follow-up experiment where we took out communication with the alien from the cover story, making the two speaker conditions more directly comparable.

## EXPERIMENT 2

This experiment was conducted to address the potential concern that the effect in Experiment 1 may have arisen not from the speaker identity manipulation but from the alien cover story in the child speaker condition.

This experiment also has a larger sample size than Experiment 1 (80 participants per condition in Experiment 2 vs. 40 in Experiment 1) so that the effect of speaker can be robustly detected since it may be somewhat smaller if the difference between the speaker conditions in Experiment 1 was driven in part by the mention of an alien in the cover story in the child speaker condition.

### Participants

160 native speakers of English, 80 per speaker condition, with an approval rating of at least 95% who did not participate in the first experiment were recruited via the crowdsourcing platform Prolific. 19 participants (7 in the adult speaker condition and 12 in the child speaker condition) were excluded because their average target rating on the unambiguous trials was below 80, suggesting that they may have misunderstood the task or randomly clicked through the experiment. 6 participants (1 in the adult condition and 5 in the child condition) were excluded because their reported strategy suggested that they had misunderstood the experiment. 1 participant in the adult speaker condition was excluded because on 6 out of 8 critical trials they assigned a 100%-rating to the distractor, suggesting that they were applying a very different strategy which does not correspond either to literal or to pragmatic responding (odd-one-out, e.g., message “red” means the only *non-red* object). New participants were recruited in their place, resulting in 80 participants per condition.

### Design

#### Materials.

Materials and setup of the experiment were identical to Experiment 1, except for the instructions introducing the speaker.

Participants in the child speaker condition were told, as before, that we had conducted a study with 4-year-old children at a local kindergarten. Instructions that were allegedly given to the children mirrored those in the adult condition but used simpler language and shorter sentences. Additionally, on the instructions screen, a picture of a 4-year-old child (daughter of one of the authors) was added, showing a computer screen with three shapes corresponding to an unambiguous speaker trial and four cards with the possible messages. The child is holding up the message with the correct answer.

On the actual listener trials where participants needed to give ratings, we included a picture of the same child looking at the four messages, where the screen with the trial which the child is solving is not visible.

This was done in order to help participants imagine a 4-year-old child. Before designing this experiment, we also ran a version where we did not include pictures of a child but only the simplified instructions. In that version of the experiment, we observed a trend in the same direction as in Experiment 1 but neither the effect of speaker condition nor the interaction of speaker condition with trial type showed strong evidence for an effect, as the 95% credible intervals for both included zero. We regard that as a failed manipulation and hypothesize that simply mentioning that the speaker is a child may not be enough for the listener to take their perspective and imagine how the child would do the task. We expect that the cover story about interaction with an alien in Experiment 1 achieved the purpose of making it easier to imagine a 4-year-old and making it clear that the speaker was a small child. We hypothesize that including pictures of an actual 4-year-old will achieve that purpose while eliminating the potential confound of an alien interlocutor.

In the adult speaker condition, we also rephrased the instructions introducing the speaker slightly to make them more clear and closer to the child speaker condition. The instructions, along with the images used in the child speaker condition, can be found in the Appendix.

### Results

We again first inspect the average probability (out of 100 points) assigned to the target for each speaker type. The bottom panel of Figure 3 shows the same pattern as in Experiment 1: participants are at ceiling on unambiguous trials and at chance on ambiguous ones, suggesting that they understood the experiment and that they considered a 4-year-old to be capable at least of feature matching. On critical trials, the average target probability is 70.7 (*SE* = 1.01) in the adult speaker condition and 62.2 (*SE* = 0.85) in the child speaker condition. In the adult speaker condition, target probability is nearly identical to that in Experiment 1 (70.8 vs. 70.7), while in the child speaker condition, it is somewhat higher (62.2 vs. 57.3). This seems to suggest that the effect of speaker condition persists but that it is somewhat smaller than in Experiment 1, possibly due to the fact that in Experiment 1 it was partially driven by the mention of the alien in the cover story.

To verify this effect, we fit a Bayesian regression model in the same way as for Experiment 1. The results are reported in Table 2. We can see that Experiment 2 closely replicates Experiment 1. Again, much lower ratings were assigned to the target on critical trials compared to unambiguous trials ($\hat{\beta }$ = −16.19, 95% CrI = [−17.62, −14.79]). Likewise, like in Experiment 1, there is a small but robust effect of trial number, suggesting that participants on average became slightly better over the course of the experiment ($\hat{\beta }$ = 0.17, 95% CrI = [0.10, 0.24]). There was no effect of message type or target position.

The main effect of interest, the effect of speaker, persists and the credible interval does not include 0 ($\hat{\beta }$ = 1.90, 95% CrI = [0.41, 3.39]). The estimate for the effect of speaker is smaller than in Experiment 1 (1.90 vs. 3.37) but it does fall inside the 95% CrI for the estimate of the effect in Experiment 1. Similarly, there is an interaction of speaker and condition ($\hat{\beta }$ = 1.83, 95% CrI = [0.42, 3.27]), which is again smaller than in Experiment 1 (1.83 vs. 3.13) but does fall inside the 95% CrI for the estimate in Experiment 1. This provides more evidence for the effect of speaker identity on the derivation of pragmatic inferences.

**Figure 4.**
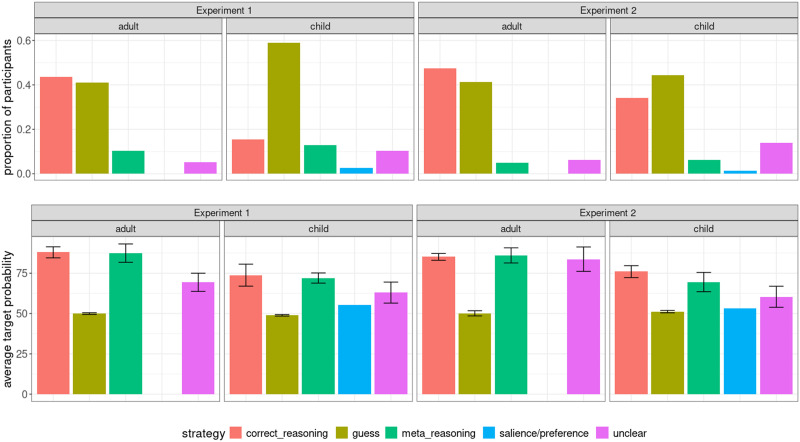
Frequency of each strategy tag per speaker condition and the corresponding target probability for that tag in the two experiments.

Next, we look at the annotations of participants’ strategies, shown in the right panel of Figure 4. As in Experiment 1, there are quite a few *guess* responses in both conditions (33 out of 80, 41.2%, in the adult condition, and 35 out of 80, 44.3%, in the child speaker condition), suggesting that even in the adult condition, there were some participants who were behaving like literal listeners themselves. While in Experiment 1 there was a much higher proportion of *correct_reasoning* responses in the adult speaker condition compared to the child speaker condition, in Experiment 2 this pattern persists but it is much less pronounced (47.5% vs. 34.2%), suggesting that the effect may be less categorical and more graded.

There are again a few *meta_reasoning* responses, 4 in the adult speaker condition and 5 in the child speaker condition, corresponding to making a less strong inference when considering the speaker. In the adult speaker condition, the following explanations are provided: “I can’t discount that they didn’t act overly quickly without thinking”, “They may not have noticed it”, “They may have rushed and not noticed” and “There’s a small chance the participant giving the answer wasn’t paying attention”. In the child speaker condition, the reasons given were “I don’t know how good kids are at thinking logically like that”, “This depends on the mental age of the child”, “I have some doubt in the child’s ability to make this more complex decision so I’ve left 25% of room for doubt”, “There’s always a chance as a 4-year-old the will have just picked the shape”, “[If they had wanted to refer to the competitor], they would (probably, if their reasoning skills were advanced enough) have chosen the triangle message”. There seems to be a distinction that in the adult speaker condition, when people reason about the speaker, they draw a less strong inference because they account for the person rushing or not paying attention in the moment, whereas in the child speaker condition, the discounting happens because of uncertainty about the child’s *ability* to reason optimally.

Like in Experiment 1, there is only one explanation that falls into the *salience*/*preference* category, also in the child speaker condition. It is “the blue seems more striking so I thought maybe they would have noticed it more than the red”. Interestingly, this explanation is exactly the opposite of the salience explanation in Experiment 1, which supposed that children may have a preference for red, suggesting that people may differ in their perception of salience.

When we examine average target ratings, we find, like in Experiment 1, that for the same strategy tag, the associated probability is higher in the adult speaker condition than in the child speaker condition: 85.2 (*SE* = 2.13) vs. 76 (*SE* = 3.69) for *correct_reasoning*, 86.1 (*SE* = *4.70*) vs. 69.5 (*SE* = *5.96*) for *meta_reasoning* and 83.7 (*SE* = *7.57*) vs. 60.4 (*SE* = *6.53*) for *unclear*. This suggests that even when people make a pragmatic inference in the child speaker condition, they are less certain about it and assign a higher probability to the child not choosing an optimal message than to an adult not doing so.

### Discussion

Experiment 2 successfully replicated the effect of speaker identity on the strength of pragmatic inferences while removing the potential confound introduced by the mention of an alien in the instructions of the child speaker condition in Experiment 1, with a larger sample size.

The estimates of the effects of interest (main effect of speaker and interaction of speaker with trial type) were smaller than in Experiment 1 but fell within the 95% CrI of the estimates for those effects from Experiment 1. This suggests that a part of the effect in Experiment 1 may have been driven by the fact that people were interpreting children’s messages less pragmatically because the child was said to be communicating with an alien. Therefore the estimates of the effect of speaker identity and the interaction of speaker identity with trial type obtained in Experiment 2 are likely to be more accurate.

**Figure 5.**
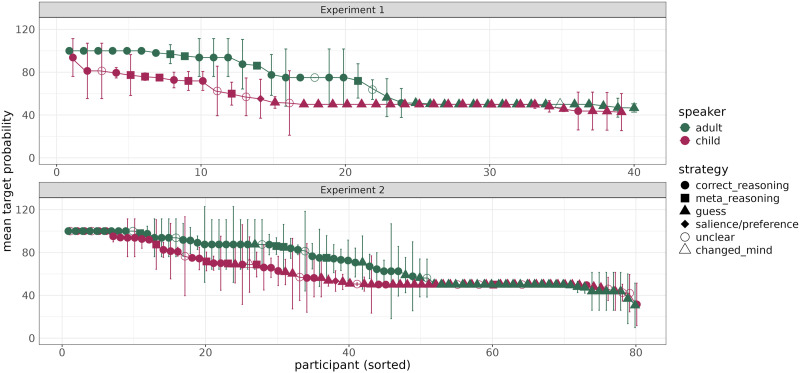
Individual participants’ average target ratings by speaker condition in the two experiments, sorted from high to low, with reported strategies.

The effect that we observed is a population-level effect. If we examine the performance of individuals in each condition, we again observe a lot of variability. Figure 5 shows mean probability assigned to the target on critical trials by individual participants in the two speaker conditions, sorted from high to low. This individual variability indicates that different people are likely to have different beliefs about rationality of speakers, and possibly also that people utilize the scale differently.

Is the observed effect categorical or gradient in nature? In other words, do fewer people in the child speaker condition derive an inference, or do comparably many people derive a less strong inference? Examining individual participants’ performance (Figure 5) suggests that it is likely to be both: more participants consistently assign a 50% probability to the target, consistent with literal responding, in the child speaker condition than in the adult speaker condition, but there is also a gradient component, where people in both conditions draw an inference but it is less strong in the child speaker condition, as indicated by the line corresponding to individual participants’ performance in the adult speaker condition being above the line for the child speaker condition. Additional evidence for a less strong inference in the child speaker condition comes from lower target ratings corresponding to the reported *correct_reasoning* and *meta_reasoning* strategies in the child speaker condition compared to the adult speaker condition.

We also see that, even in the adult speaker condition, there are some people who assign the probability of near 50% to the target, consistent with a literal interpretation. We do not expect people to believe that another adult is unable to reason about alternatives. Therefore, those people likely had no internal model of the speaker and just interpreted messages literally themselves, which corresponds to the literal listener RSA model *L*_0_. This is consistent with Franke and Degen (2016), who found that a literal listener model fit the data of a subset of their participants best.

## GENERAL DISCUSSION

In this study, we asked whether people adjust their interpretation of ambiguous messages based on how rational they expect the speaker to be. In two experiments, we asked participants to play a reference game where their task was to identify the referent of a message. We manipulated the identity of the sender of the message: participants were told that the sender was either another adult or a 4-year-old child. We found that participants were more likely to interpret an ambiguous message pragmatically if they thought that the speaker was another adult than if it was a child.

We observed that there is likely both a categorical and a gradient component to this effect: more people appear to not derive inference in the child speaker condition, and participants in the child speaker condition who do derive an inference derive a less strong inference than those in the adult speaker condition.

This finding contributes to the small but growing body of work that suggests that listeners track properties of their interlocutors and adjust the interpretations of their utterances accordingly.

We now return to the question of how this reasoning about the speaker can best be captured in the Rational Speech Act framework. In both the child and the adult speaker conditions, participants appear to belong to a mixture of reasoning types. See the Appendix for an additional analysis which compares experimental data to simulated participants drawn from a normal distribution with the mean and standard deviation from each speaker condition, which represents the assumption that all participants in each speaker condition used the same strategy and that individual differences are due to sampling chance, and chance. It illustrates that, in both speaker conditions, the differences between subsets of participants are more extreme than would be expected under a unimodal normal distribution, suggesting that multiple strategies were used in each speaker condition.

In both speaker conditions, there are participants who assigned the probability of about 50% to the target, consistent with the predictions of the *L*_0_ RSA model. Since we do not expect adults to assume that other adults are completely unable to reason rationally, we assume that all participants who assigned a rating near 50% to the target in the adult speaker condition are literal *L*_0_ listeners who do not have an internal model of the speaker and were not able to solve the task pragmatically themselves.

In the child speaker condition, a higher proportion of participants assigned an equal probability to the target and to the competitor than in the adult speaker condition. There are two possible explanations for this, which are not mutually exclusive.

First, it is possible that some such literal-looking responding in the child speaker condition comes from reasoning about the child speaker and deciding that the speaker is literal, therefore deciding not to derive the inference. Assigning a rating of 50% is consistent with the predictions of *L*_0_ and not *L*_1_ (or, equivalently, $L_{2}^{\text{belief}-\text{driven}}$ with *λ* = 0) reasoning about *S*_0_, because *L*_1_ will automatically consider the available messages and their respective probabilities and assign the probability of at least $\frac{2}{3}$ to the target, as discussed in Background: Rational Speech Act Models section. Indeed, Mayn et al. (2024) found in a similar paradigm that when participants were told that they were interacting with an explicitly literal speaker (a computer program *basic_message_speaker*), participants themselves behaved consistently with predictions of *L*_0_ and not *L*_1_, failing to consider the probabilities of alternative messages. Therefore, some of the 50% responding in the child speaker condition may result from deciding that the child is a literal speaker but making an error in probabilistic computations. We think that this is possible but not very likely since all instances of participants reporting a *meta_reasoning* strategy corresponded to higher target ratings, i.e., participants appear to be relatively certain that children are fairly capable of behaving as pragmatic speakers, whereas an average target probability of 50% corresponds to the reported strategy *guess*.

The second and, in our view, more likely explanation for there being more literal-appearing responders in the child speaker condition is that the situational context of reasoning about a child is more conducive to an *L*_0_-level reasoning setting. It is known that we can process information more or less deeply depending on the context. Daniel Kahneman (2011) famously framed these distinct processing mechanisms as two systems, a quick, heuristic System 1, and a slower System 2 that engages in deeper processing. In language comprehension research, the influential “good enough processing” account states that in everyday language processing comprehenders often rely on heuristics and compute more coarse-grained, “good enough” representations unless the task requires them to expend more effort and create a more fine-grained interpretation (Ferreira & Patson, 2007). In line with these accounts, it could also be hypothesized that people, as comprehenders and reasoners, have different reasoning settings, a more shallow setting that approximately corresponds to the *L*_0_ listener model and a deeper reasoning setting that corresponds to the *L*_2_ model. It could be, then, that the conversational context may motivate the comprehender to shift into one setting or the other: in the case when the interlocutor is another adult, people may be more likely to expect intentionality and cooperativity of their interlocutor, which motivates them to reason more deeply about possible intentions behind the sent message. Under this account, we could model the categorical differences between the two conditions in Figure 5 as there being more *L*_0_s and fewer *L*_2_s in the child speaker condition compared to the adult speaker condition, which could explain the categorical part of the observed effect. Future work could further explore this account and explore communicative settings which may cause a comprehender to shift into a deeper or a more shallow reasoning setting.

The gradient part of the observed effect, i.e., that participants in both conditions derive a pragmatic inference but it is stronger in the adult speaker condition, can be modeled using the belief-driven pragmatic listener model $L_{2}^{\text{belief}-\text{driven}}$ proposed in Background: Rational Speech Act Models section, whose beliefs about whether the speaker will select the optimal message are governed by the weight parameter *λ*. In the adult condition, the *λ* would be higher, reflecting higher certainty that the speaker is pragmatic and capable of selecting the optimal message.

Interestingly, the effect of speaker on the derived pragmatic inferences only emerged when the instructions included a visual which made the child speaker setting more salient—a drawing of an alien in Experiment 1 and a photo of a 4-year-old in Experiment 2. Before designing Experiment 2, we ran a version of the experiment where we took out the alien communication game cover story from the child speaker condition and simply told participants that the messages came from a 4-year-old. The effect was much smaller and less evident in that version, suggesting that this kind of perspective taking is difficult in the abstract—when not immersed in the situation of actually communicating with the speaker directly—and requires sufficient salience of the speaker’s characteristics to be able to take their perspective. This is consistent with findings about other perspective-taking phenomena, such as visual perspective taking in a director-matcher task (Keysar et al., 2000) and interpretation of sarcastic messages (Epley et al., 2004).

Reasoning needed to solve the critical trials in this study relies on reasoning about messages which are available and unavailable to the speaker: if both features of the target were expressible as messages, the message on critical trials would be fully ambiguous between the target and the competitor. One could argue that having not all features expressible as words or utterances is an artificial assumption which does not hold for the way we communicate using language. That is likely the case. However, this derivation process needed to resolve the ambiguity—considering the alternative meanings and that there might be better utterances to express an alternative meaning—is very similar to the one that is assumed for implicatures in language (e.g., Noveck, 2001). Also, there may be situations in linguistic communication where there is another way to express a feature of a referent but it is less accessible because it is, for example, much less frequent. In this study, as discussed in Design section, we decided to use stimuli corresponding to the simple condition from Franke and Degen (2016), which relies on some features not being expressible as messages, because Franke and Degen (2016) and Mayn and Demberg (2023) report less pragmatic performance in the complex condition, where all object features are expressible, than in the simple condition. If the task is too complex for participants to solve themselves, then they would be unlikely to additionally reason about the speaker. Future work may extend our findings by conducting similar studies in a setting where all object features are expressible. For instance, one could have an adult and a child record underinformative sentences and compare whether people draw less strong pragmatic inferences when hearing an utterance spoken by a child.

## ACKNOWLEDGMENTS

We would like to thank the anonymous reviewers for their thoughtful comments and John Duff for his insights and helpful discussions.

## FUNDING INFORMATION

This work was supported by the European Research Council (ERC) under the European Union’s Horizon 2020 Research and Innovation Programme (ERC starting grant “Individualized Interaction in Discourse”, grant agreement No. 948878).

## AUTHOR CONTRIBUTIONS

Alexandra Mayn: Conceptualization; Data curation; Formal analysis; Investigation; Methodology; Writing – original draft; Writing – review & editing. Jia E. Loy: Conceptualization; Methodology; Writing – review & editing. Vera Demberg: Conceptualization; Methodology; Writing – review & editing.

## DATA AVAILABILITY STATEMENT

Preregistrations, data and analysis scripts for this study are publicly available in the following repository: https://osf.io/f5nmv/.

## Note

^1^ It is, in fact, more common in the literature to refer to the pragmatic listener model which reasons about *S*_1_ as *L*_1_ and not *L*_2_. However, we call it *L*_2_ here to distinguish it from a listener model reasoning about a literal speaker *S*_0_. This latter model we call *L*_1_, following the notation from Franke and Degen (2016), who also make this distinction.

## Figures and Tables

**Figure 6.**
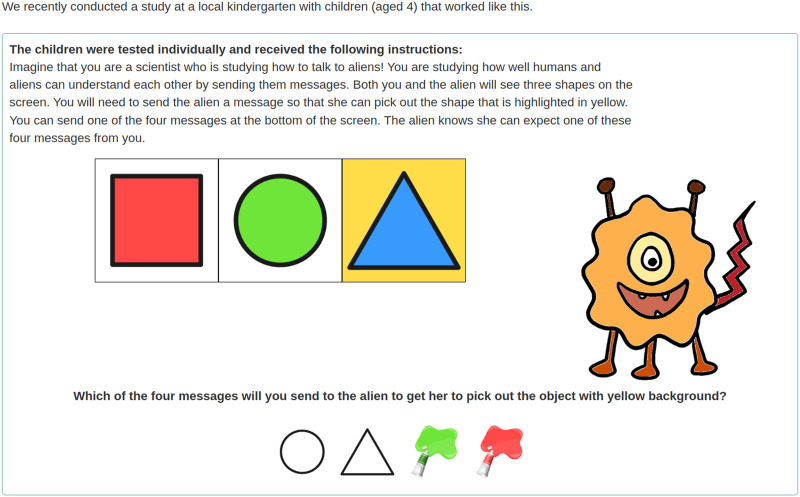
Instruction screen which introduced the speaker in the child speaker condition in Experiment 1.

**Figure 7.**
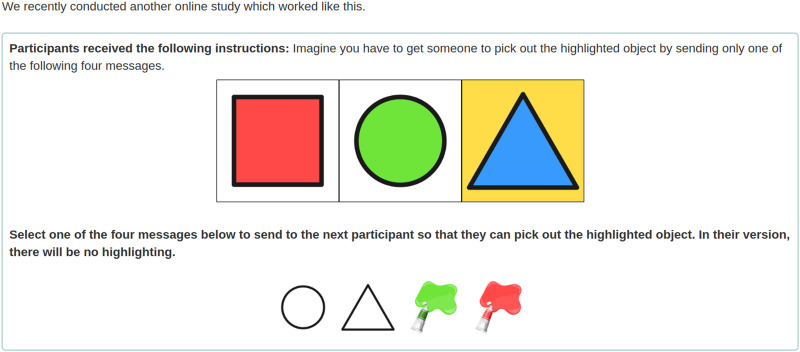
Instruction screen which introduced the speaker in the adult speaker condition in Experiment 1.

**Figure 8.**
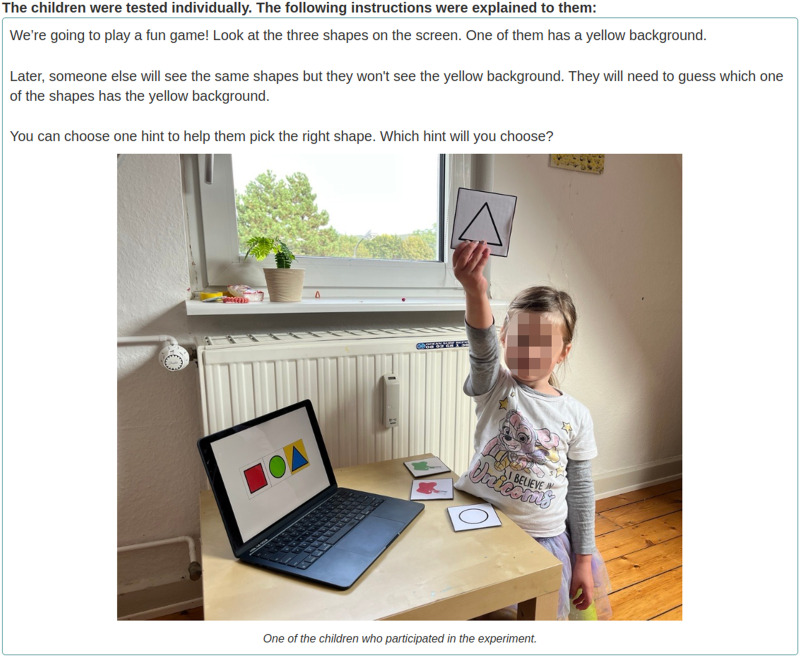
Instruction screen which introduced the speaker in the child speaker condition in Experiment 2.

**Figure 9.**
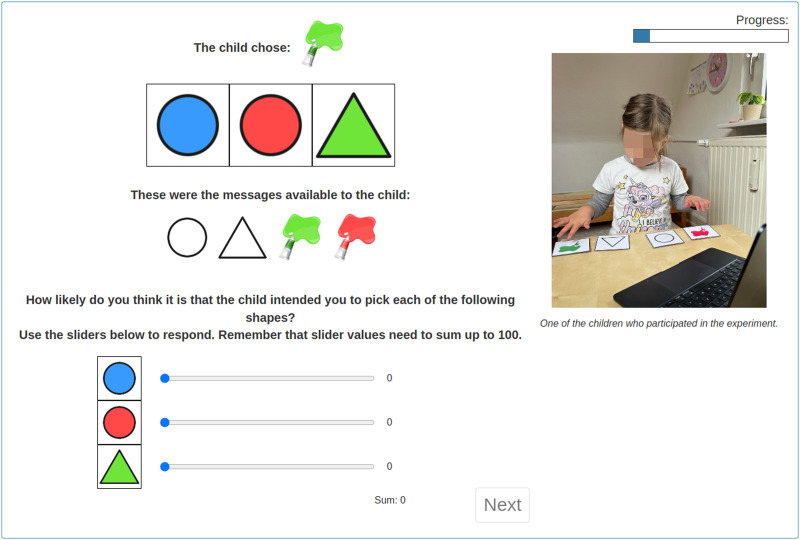
One of the trials in the child speaker condition in Experiment 2. The photo stayed the same for all trials.

**Figure 10.**
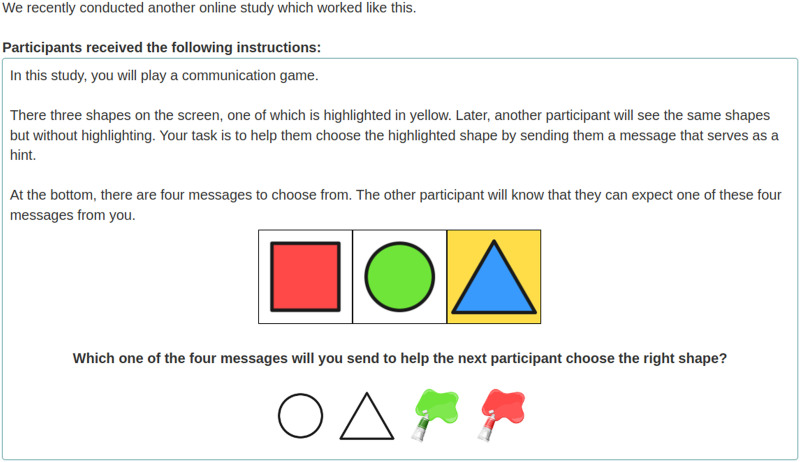
Instruction screen which introduced the speaker in the adult speaker condition in Experiment 2.

**Figure 11.**
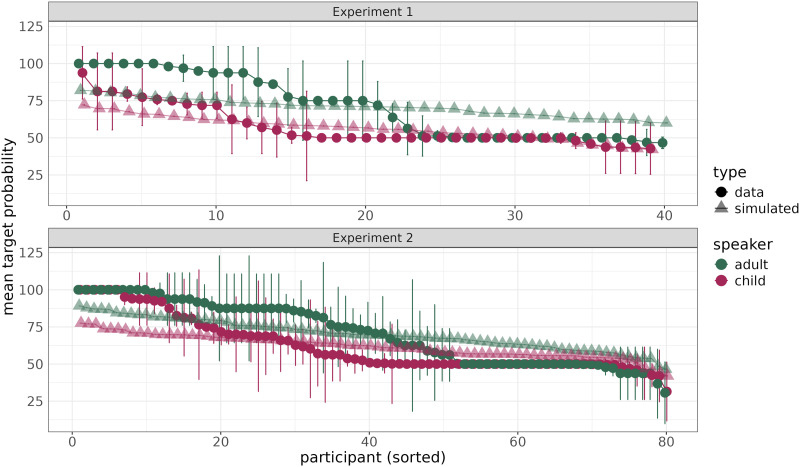
Individual participants’ average target ratings by speaker condition, sorted from high to low. The darker points are the data obtained from the experiment and the lighter points are simulated participants drawn from a normal distribution with the corresponding population-level mean and variance.

## APPENDIX

### A: Experiment Instructions

Below are the instruction screens that introduced the speaker and the speaker’s task in both conditions in the two experiments.

#### Experiment 1.

**Child speaker condition:** Instruction screen that introduced the speaker in the child speaker condition in Experiment 1 is shown in Figure 6.

**Adult speaker condition:** Instruction screen which introduced the speaker in the adult speaker condition in Experiment 1 is shown in Figure 7.

#### Experiment 2.

**Child speaker condition:** The child’s face in the photographs is blurred for privacy reasons. It was not blurred in the experiment.

Instruction screen which introduced the speaker in the adult speaker condition in Experiment 2 is shown in Figure 8.

Additionally, a picture where the child is looking at the possible messages was added to the screen with sliders for every trial. The same photograph stayed on the screen for all trials. One of the trials is shown in Figure 9.

**Adult speaker condition:** In Experiment 2, instructions introducing the speaker in the adult speaker condition were rephrased slightly to be more clear and closer to those in the child speaker condition. The instructions are shown in Figure 10.

### B: Annotation Guidelines

The following annotation scheme was used for annotating the reported strategies. Below are the definitions of each tag, as well as examples from the data, slightly edited for clarity.

Annotation tag: ***correct_reasoning***

▪ Definition: The reported strategy describes counterfactual reasoning—if the speaker had meant X, they would have said Y—formalized by a pragmatic RSA listener model.
▪ Examples
  – “The child could have picked the triangle to direct me to the triangle but picked red instead so I believe they wanted to direct me away from the triangle to the square.”
  – “There was a triangle they could have used, but not a square, so it’s likely it was the green square they wanted me to pick.”
  – “They are definitely indicating a circle. There is no colour swatch for the blue circle so by process of elimination the white circle indicates the blue one.”

Annotation tag: ***meta_reasoning***

▪ Definition: The reported strategy explicitly mentions what the correct pragmatic reasoning would be but also expresses uncertainty about whether the speaker behaved optimally.
▪ Examples
  – “Because it has to be a circle, and there isn’t an option to choose blue. So the empty circle would be the correct choice from the child if the blue circle had the yellow background, but I don’t know how good kids are at thinking logically like that.”
  – “I have some doubt in the child’s ability to make this more complex decision so I’ve left 25% in room for doubt, and 75% likelihood for the blue triangle, as the child didn’t have a blue paint option to differentiate the two triangle options.”
  – “The messages available don’t have a blue colour to distinguish the different circles if the blue one was highlighted, therefore it is most likely the blue circle is the right answer. There is a small chance the participant giving the answer wasn’t paying attention, therefore I put a 1% chance on the other circle.”

Annotation tag: ***guess***

▪ Definition: Participant says that there is no way to choose between two objects (target and competitor) since both objects have the feature expressed by the message.
▪ Examples
  – “The clue is a triangle, there are two of them so it’s a 50/50 split.”
  – “The clue is green so it is equally likely to be the triangle or the square.”
  – “Previous participant chose red. I split the slider on the two given red shapes 50/50.”

Annotation tag: ***salience*/*preference***

▪ Definition: The reported strategy suggests that the speaker must have been referring to a certain object because a feature it has (e.g., color, position) is salient or is preferred by the speaker.
▪ Examples
  – “The child would have picked the first shape with a green colour which in this case is a square.” (object position)
  – “The choice was one of the two circle shapes - the blue seems more striking so I thought maybe they would have noticed that more than the red.” (color salience)
  – “Triangles may be more popular.” (shape preference)

Annotation tag: ***unclear***

▪ Definition: It is unclear from the answer what strategy the participant was using or what they meant.
▪ Examples
  – “Based on how I thought the child reasoned, for each one.”
  – “It was based on the probability of the right colour or shape.”
  – “I used the rule of deduction.”

Annotation tag: ***misunderstood_instr***

▪ Definition: The reported strategy suggests misinterpreting the instructions that the speaker could not utter a certain message, e.g., blue, as meaning that they could not refer to an object if it had that feature (e.g., a blue triangle cannot be the intended referent since “blue” is not an available message).
▪ Examples
  – “There was no square available in the messages available to the child so it had to be the green triangle” (As a reason to rule out the square as a possible referent)
  – “The child was not given a square message, only a circular one, which is green.” (As a reason to rule out the square as a possible referent)
  – “The child chose a circle, and only red was available to match.” (As a reason to rule out the blue circle as a possible referent)
▪ Notes: Participants whose reported strategy fell into the *misunderstood_instr* category were excluded from all analyses because their reported strategy indicated that they misunderstood the instructions and were doing a fundamentally different task.

Annotation tag: ***changed_mind***

▪ Definition: Participant changed their mind upon reflection, leading to their strategy not matching the ratings they provided using sliders.
▪ Examples
  – “I’ve made an error to be honest, after carefully thinking just now it should be blue.”
▪ Notes: Participants whose reported strategy fell into the *changed_mind* category were excluded from the annotation analysis only, but included in the main regression analysis.

### C: Regression Model Priors

The following Bayesian regression model was fit to the data using the *brms* package in R:

$$\text{targetRating}∼\text{speaker}*\text{trialType}+\text{trialID}+\text{msgType}+\text{targetPos}+\left(1+\text{trialType}+\text{msgType}+\text{trialID}|\text{participantID}\right)+\left(1|\text{itemID}\right)$$

For all effects, we set wide weakly informative priors in order to avoid biasing the model. Below we list the priors for all effects with a brief rationale.

**Fixed effects:**

▪ Intercept
  – Prior: Normal(87.5, 12.5)
  – Rationale: We expect the ratings on unambiguous trials to be at ceiling (i.e., near 100) for both speaker conditions, and on critical trials, the plausible range of the ratings is 50–100 (50 corresponding to literal responding and 100 corresponding to perfectly pragmatic responding), so we take the middle point, 75, as the hypothesized mean for critical trials. Therefore, we set the mean of the intercept to the midpoint between 75 and 100, with a wide standard deviation.
▪ Speaker
  – Prior: Normal(0, 12.5)
  – Rationale: We do not want to assume the direction of the speaker effect so we set the mean to be 0. The standard deviation is set to be 12.5 so that the whole plausible range of target ratings, 50–100 (50 corresponding to literal responding and 100 corresponding to perfectly pragmatic responding) is covered by 2 standard deviations from the mean.
▪ Trial type
  – Prior: Normal(−25, 12.5)
  – Rationale: We expect target ratings on unambiguous trials to be at ceiling, i.e., near 100. Critical trials can plausibly take up the space between 50 and 100, so we take the midpoint as the mean with the standard deviation of 12.5, so that the decrease of ratings of 0 to 50 points compared to the unambiguous trials is covered by 2 standard deviations from the mean.
▪ Speaker: Trial type interaction
  – Prior: Normal(0, 12.5)
  – Rationale: Here, again, we do not want to assume the direction of the interaction and allow for the range 50–100 to be covered within two standard deviations.
▪ TrialID
  – Prior: Normal(0,5)
  – Rationale: We assume this to be a very small effect based on the results of previous studies in similar paradigms (e.g., Franke & Degen, 2016; Mayn & Demberg, 2023).
▪ Message type
  – Prior: Normal(0, 5)
  – Rationale: This is a covariate which we expect to have a very small effect based on the findings of previous studies.
▪ Target position
  – Prior: Normal(0, 5)
  – Rationale: This is also a covariate which we also expect to have a very small effect based on the findings of previous studies.

**Random effects:**

▪ Standard deviation of random effects for participant
  – Prior: student_t(3, 0, 12.5)
  – Rationale: We set a wide prior with a large scale parameter of 12.5 to account for potential variability in participant effects.
▪ Standard deviation of random effects for item
  – Prior: student_t(3, 0, 2.5)
  – Rationale: We do not expect there to be much variability between items based on previous work in the reference game paradigm with similar items (e.g., Mayn & Demberg, 2023), resulting in a smaller scale parameter of 2.5.
▪ Residual standard deviation
  – Prior: student_t(3, 0, 2.5)
  – Rationale: The scale parameter of 2.5 reflects the fact that we do not expect wide variation in residuals.
▪ Correlation structure
  – Prior: LKJ(2)
  – Rationale: We set a weakly informative prior allowing for moderate correlation of random effects.

### D: Derivation of RSA Model Predictions

Let’s show what predictions the proposed listener model $L_{2}^{\text{belief}-\text{driven}}$ makes for our experiment in different cases. We will use the example from Figure 2 but we will not consider the distractor since it is not relevant to the derivation of the inference. Therefore, we will consider two referents (red square and red triangle) and two messages (“red” and “triangle”).

The model is defined as follows:

$$L_{2}^{\text{belief}-\text{driven}}\left(o|u\right)\propto S_{\text{weighted}}\left(u|o\right)\cdot P\left(o\right)$$

$$\text{where}\;S_{\text{weighted}}\left(u|o\right)\propto \lambda \cdot S_{1}\left(u|o;\alpha \to \infty \right)+\left(1-\lambda \right)\cdot S_{0}\left(u|o\right)\;\text{and}\;0\leq \lambda \leq 1$$

Let’s derive the model’s behavior at endpoints, *λ* = 0 and *λ* = 1, as well as at an intermediate point, *λ* = 0.5.

#### Listener Who Is Certain That the Listener is Literal: *λ* = 0.

When *λ* = 0, the *S*_*weighted*_(*u*∣*o*) equation simplifies to *S*_0_(*u*∣*o*).

**Table:**

	“red”	“triangle”
red sq.	1	0
red tr.	0.5	0.5

$L_{2}^{\text{belief}-\text{driven}}$ with *λ* = 0 is essentially *L*_1_. Assuming uniform prior probability over objects, *L*_1_(*o*∣*u*) ∝ *S*_0_(*u*∣*o*). To obtain the listener probability distribution, we transpose the *S*_0_ matrix and normalize it:

**Table:**

	red sq.	red tr.			red sq.	red tr.
“red”	1	0.5	→	“red”	**0.66**	0.33
“triangle”	0	0.5		“triangle”	0	1

We can see that, despite the fact that the speaker is fully literal, *L*_1_ correctly prefers the target (red square) to the competitor (red triangle) because of reasoning about the prior probabilities of alternatives.

#### Listener Who Is Certain That the Speaker is Fully Pragmatic: *λ* = 1.

When *λ* = 1, the listener model *S*_*weighted*_(*u*∣*o*) simplifies to *S*_1_(*u*∣*o*; *α* → ∞).

*S*_1_ reasons about literal listener *L*_0_. *L*_0_(*o*∣*u*) interprets the messages literally and assigns equal probability to all literally true referents. Assuming uniform prior probability of objects, *L*_0_(*o*∣*u*) ∝ [[*u*]], where [[*u*]] is a boolean function evaluating whether the utterance is literally true of the object. Thus, for our example, *L*_0_(*o*∣*u*) is:

**Table:**

	red sq.	red tr.
“red”	0.5	0.5
“triangle”	0	1

The speaker is then defined as *S*_1_(*u*∣*o*; *α* → ∞) ∝ *exp*(*α* · (log *L*_0_(*o*∣*u*)), assuming zero utterance costs for simplicity. The speaker temperature parameter *α* controls the degree to which the speaker maximizes their utility. Since we want the model to represent a speaker who always sends the pragmatically optimal message, we set *α* → ∞.

To obtain the *S*_1_ distribution, we transform the *L*_0_ matrix and calculate *exp*(*α* · log *L*_0_) for *α* → ∞:

**Table:**

	“red”	“triangle”			“red”	“triangle”
red sq.	0.5	0	→	red sq.	1	0
red tr.	0.5	1		red tr.	0	1

As a result, the fully pragmatic speaker *S*_1_(*u*∣*o*) always uses the message “red” to refer to the red square and the message “triangle” to refer to the red triangle.

Finally, to obtain the listener distribution *L*_2_(*o*∣*u*), we transpose the *S*_1_(*u*∣*o*) matrix (which is already normalized):

**Table:**

	red sq.	red tr.
“red”	**1**	0
“triangle”	0	1

Thus, at *λ* = 1, the listener will be perfectly certain that “red” refers to the red square.

#### Uncertain Listener: *λ* = 0.5.

A listener whose lambda is in between the endpoints will have uncertainty about whether the speaker is literal or pragmatic and will weigh the two speaker models equally.

We’ll show the computation for *λ* = 0.5 but the principle is the same for any intermediate *λ* value.

$L_{2}^{\text{belief}-\text{driven}}$ (*o*∣*u*) with *λ* = 0.5 will weigh the two speaker models, *S*_0_(*u*∣*o*) and *S*_1_(*u*∣*o*; *α* → ∞), equally.

To obtain *S*_*weighted*_(*u*∣*o*), we take the *S*_0_(*u*∣*o*) and *S*_1_(*u*∣*o*; *α* → ∞) matrices, multiply each matrix by *λ* = 1 − *λ* = 0.5, add them together and normalize:

**Table:**

		“red”	“triangle”				“red”	“triangle”			“red”	“triangle”
0.5 ·	red sq.	1	0	+	0.5 ·	red sq.	1	0	→	red sq.	1	0
	red tr.	0.5	0.5			red tr.	0	1		red tr.	0.25	0.75

Finally, to obtain the *L*_2_(*o*∣*u*) distribution, we transform the *S*_*weighted*_ matrix and normalize:

**Table:**

	red sq.	red tr.			red sq.	red tr.
“red”	1	0.25	→	“red”	**0.8**	0.2
“triangle”	0	0.75		“triangle”	0	1

Thus, $L_{2}^{\text{belief}-\text{driven}}$(red square∣“red”) when *λ* = 0.5 is 0.8.

With different *λ* values, the probability range of $\frac{2}{3}$ to 1 is covered.

### E: Sorted Participant Plot With Simulated Data

We hypothesize that our participants use a mix of different reasoning strategies—literal listeners as well as pragmatic listeners with different beliefs about the speaker’s rationality—as opposed to just one strategy. We ask how closely the data we obtained in the experiments aligns with normal distributions with population-level mean and variance in each speaker condition, which would represent the assumption that all participants use the same strategy and the differences between them are due to sampling error.

To answer that question, we plot actual participants’ target ratings, sorted from high to low, as well as simulated participants drawn from normal distributions with population-level mean and variance in each speaker condition (Figure 11). When we compare empirical data to the simulated data, we see that for both experiments, the alignment is not very good: there are more extreme high ratings and chance-level responses than would be predicted by a unimodal distribution that assumes that all participants used the same reasoning strategy. This suggests that the variation that we observe reflects differences in strategies, which should correspond to distinct models, as opposed to mere sampling chance.
